# Gender dimensions of youth vulnerability toward access to cigarettes in South-East Asia: Evidence from global youth tobacco survey

**DOI:** 10.3389/fpubh.2022.976440

**Published:** 2022-11-10

**Authors:** Nancy Satpathy, Pratap Kumar Jena, Venkatarao Epari

**Affiliations:** ^1^Department of Community Medicine, Institute of Medical Sciences and SUM Hospital, Siksha ‘O' Anusandhan Deemed to be University, Bhubaneswar, Odisha, India; ^2^School of Public Health, Kalinga Institute of Industrial Technology Deemed to be University, Bhubaneswar, Odisha, India

**Keywords:** access, youth, cigarettes, tobacco, global youth tobacco survey, South East Asia

## Abstract

**Background:**

Youths are lured to smoking to make them tobacco customers. Limiting access to tobacco products by youths is a proven strategy to reduce youth tobacco use. This study aimed to examine the burden of cigarette smoking and access to tobacco by youth in South-East Asia (SEA).

**Methods:**

The burden along with the physical (methods of obtaining cigarettes), financial (cigarette affordability by pocket money), and illegal (sale to minors) access to cigarettes among school-going boys and girls were examined by analyzing the Global Youth Tobacco Survey (GYTS) data (2013–2016) from seven SEA member countries. Descriptive statistics using country-specific GYTS sample weight was used to estimate parameters with 95% confidence intervals.

**Results:**

The proportion of youths reporting cigarette smoking was highest in East Timor [boys: 55.57 % (51.93–59.21) and girls: 11.35% (9.12–13.59)] and lowest in Sri Lanka [boys: 2.96% (2.91–3.0) and girls: 0%]. Smoking prevalence was higher among boys than girls. Smoking among boys and girls was positively correlated (*r* = 0.849, *p* = 0.032). The most common method of obtaining cigarettes was “buying it from a store/kiosk/street hawker” and “other sources.” Except in Indonesia, financial access was limited for most youths. Financial access had a positive but negligible influence on cigarette smoking. Despite legal restrictions on sales to minors, students could obtain cigarettes from vendors.

**Conclusion:**

Contextual cigarette smoking and access to cigarettes by youths despite the legal ban and unaffordability is a concern. Country-specific socio-cultural-economic and legal dimensions need to be examined to limit cigarette use among youths.

## Introduction

The use of tobacco kills eight million people annually (1). In many countries, the cigarette is the most commonly used tobacco product, and most smokers start cigarette smoking when they are still minor (2). Nearly nine out of 10 cigarette smokers try their first cigarette before the age of 18 years (2). Evidence shows that adult smokers with less quit intention have a history of initiating smoking in their adolescence (3). This scientific information is being used by tobacco industries to target youths for increasing sales and overall consumption.

In 2020, South-East Asia Region (SEAR) reported the highest prevalence of tobacco consumption in the world, which was around 27.9% (4). The average prevalence of tobacco use among men and women was 46 and 9.7%, respectively (5). According to the World Health Organization, tobacco use in SEAR is expected to decrease to 25.1% by 2025, following a downward trend in all regions (5). However, the consumption of multiple tobacco products is increasing, which is in sync with increased tobacco promotion by the tobacco industries in this region that seeks to build a greater consumer base among the youth (6). Therefore, reducing tobacco use among youths is the key to ending the tobacco epidemic (7). Smoking among the youths is systematically monitored through the Global Youth Tobacco Survey (GYTS) (8), which generates evidence for policy formulation and implementation of tobacco control strategies (9).

A crucial component of the comprehensive tobacco control policy is to limit the availability and demand for tobacco products to dissuade children and young people from starting to smoke. This is a mandate of those who have ratified the WHO Framework Convention on Tobacco Control (FCTC). Except for Indonesia, every other country in the SEA has signed and ratified the WHO FCTC. Besides, the enforcement of compliant FCTC comprehensive tobacco legislation differs from country to country. Indonesia is a country that is neither a signatory nor a party to the FCTC (10, 11).

Age and access limitations on the sale of tobacco products have been implemented in several nations with varying degrees of effectiveness due to resource constraints associated with enforcing these laws. The less obvious reason could be that the prohibition creates the concealed perception that tobacco consumption is an adult habit, therefore, increasing its allure among teenagers. As a result, any publicity or activities aimed at enforcing the law could make younger people more likely to want to smoke (9). Disallowing self-service displays and vending machines is seen as a more efficient and realistic measure to minimize access to tobacco products among the youth (12, 13). One further measure that falls under the purview of restricting access is the response to the introduction of new types of tobacco products onto the market (14). Consequently, one preemptive action that could be taken would be to prevent the introduction of new types of tobacco products.

Evidence demonstrates that comprehensive tobacco control strategies such as taxing, warning, and banning are required before limiting access to tobacco products (15). Before imposing any intervention to limit access to tobacco products for youth, we have to understand how and where they have access. Thus, this study examines the prevalence of cigarette use and youth vulnerability to access to cigarettes in the South-East Asia (SEA) region using GYTS data in the context of country-specific youth tobacco control policy environments.

## Methods

Context of GYTS data is from publicly available GYTS, a standardized and internationally comparable school-based survey among students aged 13–15 years from 8^th^ and 9^th^ grades. The GYTS is a part of the Global Tobacco Surveillance System that helps countries to monitor tobacco control activities as per the WHO Framework Convention on Tobacco Control (FCTC) and is funded by WHO and CDC.

The GYTS selects a representative sample of students from each nation using a two-stage cluster sampling technique. All schools are included in the sampling frame in a geographically defined area that contains any of the specified grade levels. At the initial stage, the probability that a school will be chosen is proportionate to the number of pupils enrolled in each grade. In the second stage of sampling, classes within the specified schools are selected at random. Classes within the selected schools were selected using a simple randomization technique. All students in chosen grade courses attending school on the day the survey is administered are eligible to participate in a self-administered survey. Participation is voluntary and anonymous (16). The GYTS has a set of 54 standard questions in English and local languages as per need (8). The questions mainly focus on the prevalence of smoked and smokeless tobacco among youths in schools, their accessibility to various tobacco products, their desire to quit smoking, their exposure to media and advertising, and their exposure to secondhand smoke (SHS).

Data were available for seven WHO SEA member countries. Bangladesh, Bhutan, East Timor, Indonesia, Myanmar, Sri Lanka, and Thailand conducted GYTS from 2013 to 2016 consecutively. The selection of countries was based on publicly available data. The achieved sample size ranged from 1,503 in Sri Lanka to 3,186 in Indonesia. Among the sample population total of 9,675 boys and 10,911 girls were considered with sizes ranging from 767 to 2,803 boys and 736 to 3,178 girls. The GYTS response rate ranged from 81.9% in Sri Lanka to 100% in Bangladesh (1).

## Study variables

This study examined physical access (method of obtaining cigarettes), illegal access (sales to minors), financial access (financial affordability, i.e., pocket money exceeding a pack of 20 cigarettes), and cigarette smoking. The analysis was further gender stratified, which represents a socio-cultural factor. The proportion of students reporting one or more cigarettes during the preceding 30 days of the survey was used to estimate the prevalence of current smoking (16). Students, who smoked currently, were asked about their primary mode of obtaining cigarettes. Physical access to cigarettes was assessed by the question, “During the last 30 days, how did you usually get your cigarettes?” with the following answers: “I bought them in a store, shop or from a street vendor”; “I bought them from a vending machine”; “I gave someone money to buy them for me”; “I borrowed them from someone else”; “I stole them”; “An older person gave them to me”; or “I got them some other way.” The last four options were combined to form “other” sources.

The financial access was identified by the GYTS question “During an average week, how much money do you have that you can spend on yourself, however, you want?” and “On average, how much do you think a pack of 20 cigarettes cost?” (17). In this study, when the pocket money for 7 days was adequate to purchase a pack of cigarette packs, then it was considered to have financial access to cigarettes.

Illegal access was determined when the youth had access to cigarettes from vendors despite the country's legal provisions banning sales to and sales by a minor. GYTS question “During the past 30 days, did anyone ever refuse to sell cigarettes because of your age?” along with the responses “I did not try to buy cigarettes during the past 30 days”; “Yes, someone refused to sell cigarettes because of my age”; and “No, my age did not keep me from buying cigarette” were analyzed and triangulated with country-specific tobacco control policy provision for tobacco restriction among youths.

## Data management and analysis

A descriptive statistical analysis was performed using the SPSS data processor version 21.0. Cigarette smoking prevalence was calculated as the sample number and weighted percentage. The weighting factor was incorporated into each student to account for nonresponse (by class and school) and variation in selection probability at the school and class levels. For physical access, a radar plot was present to illustrate various preferred methods for obtaining a cigarette. A dual Y-axis graph was used to describe financial access with the prevalence of cigarette smoking aged among 13–15 years school-going boys and girls in the SEA countries. The 95% confidence interval was also estimated for cigarette use prevalence. GYTS sample weight was used in the analysis. Correlation statistics were used as per the requirement.

### Ethical considerations

The GYTS data set is available in the public domain from CDC for researchers. Therefore, an ethics review was not deemed necessary.

## Results

**Figure 1:**
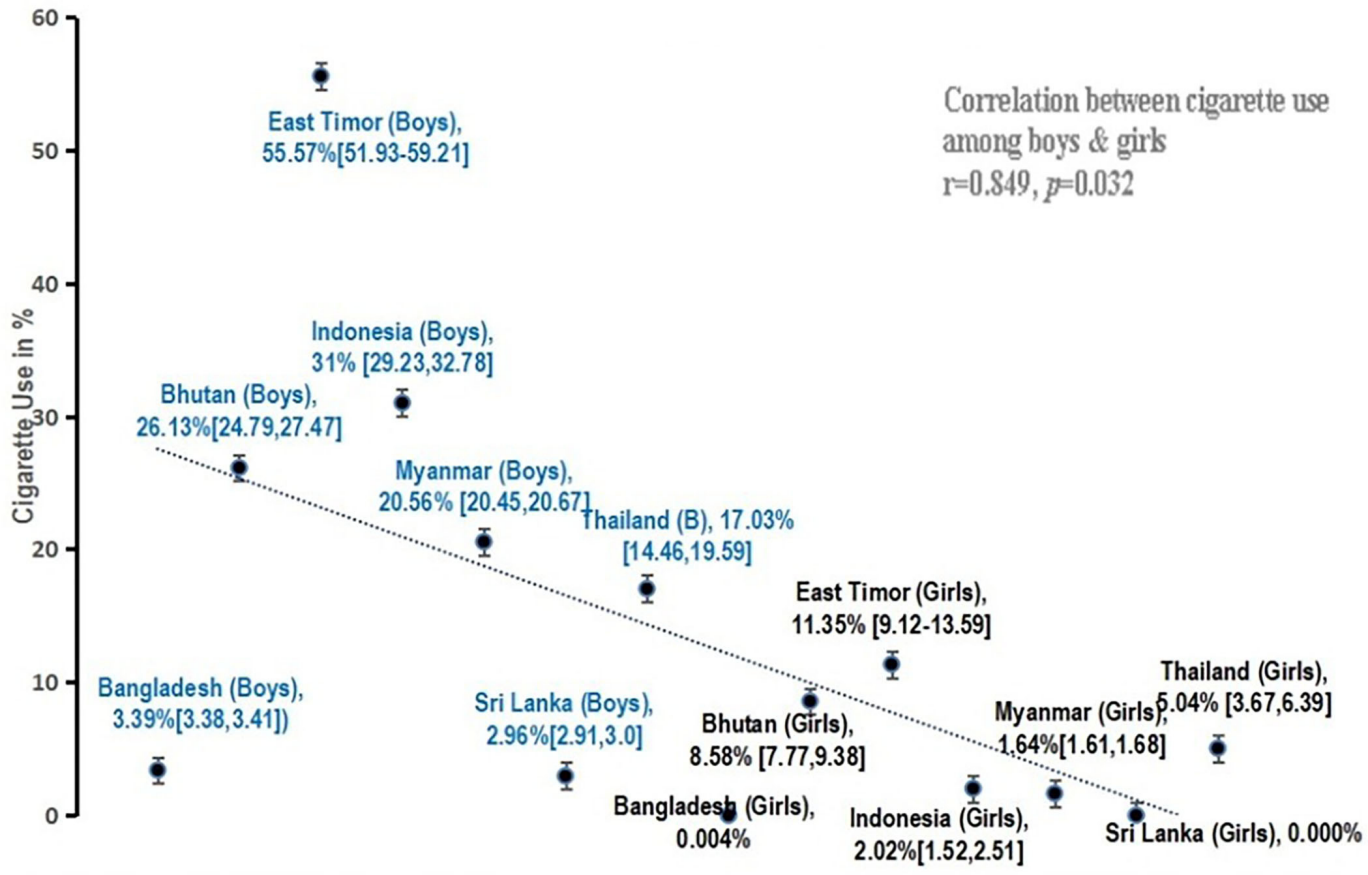
Prevalence of cigarette smoking among school-going children in seven SEA member countries.

The burden of cigarette smoking aged among 13–15 years school-going boys and girls in the SEA countries were in the range of nil to 55.57% as shown in Figure 1 with their relative positions.

Cigarette smoking was lower in Sri Lanka (boys-3%, girls-0%) and Bangladesh (boys-3.4%, girls-0.004%) among both boys and girls. The highest prevalence was seen in East Timor (boys-55.57%; girls-11.35%). The prevalence of cigarette smoking was higher among boys as compared with girls. However, there was a strong positive correlation (*r* = 0.849, *p* = 0.032) between cigarette smoking among boys and girls (Figure 1).

**Figure 2:**
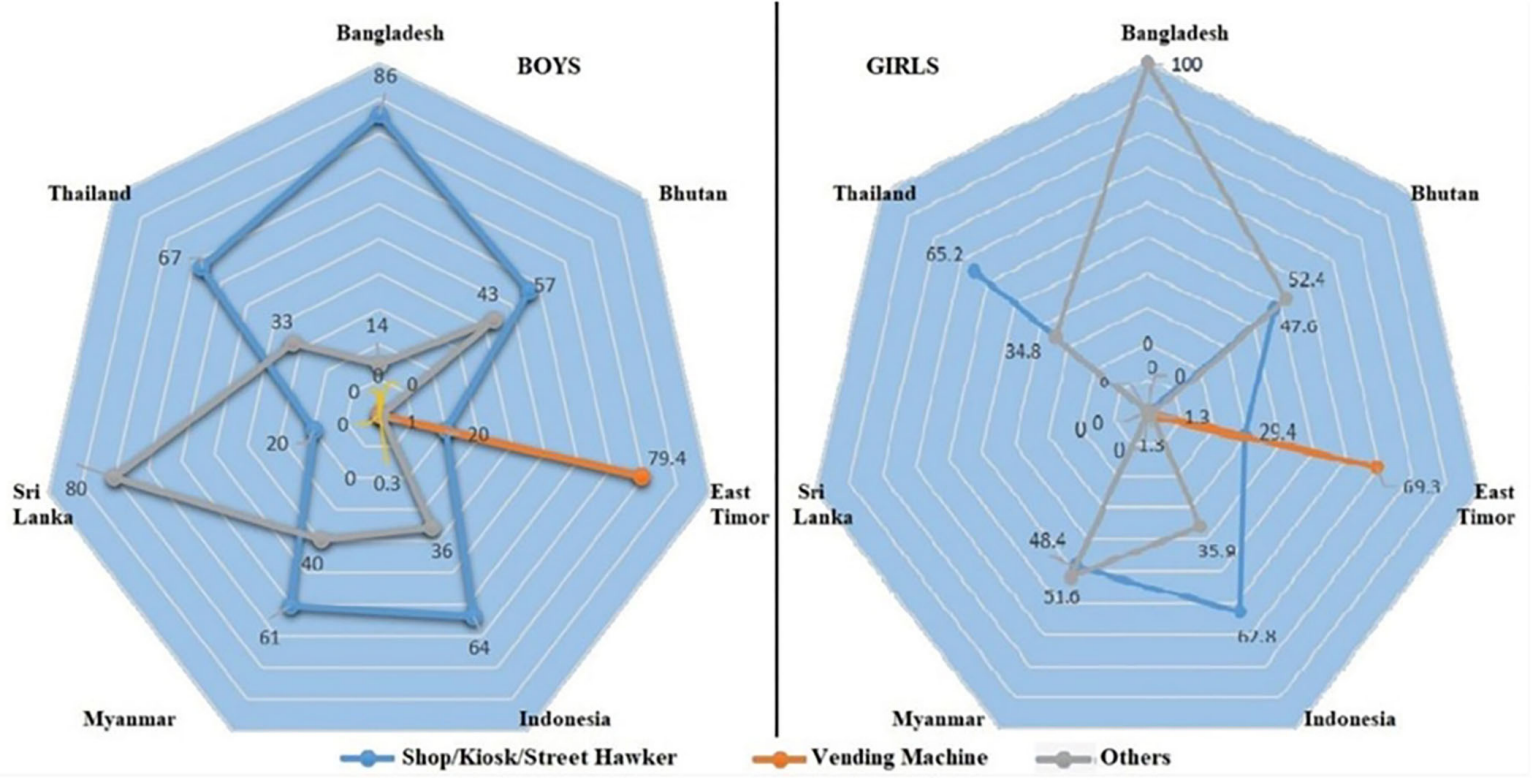
Physical access to cigarette smoking among school-going children in seven SEA member countries.

The methods of physical access to cigarettes differed from one country to another (Figure 2). The most preferred method for obtaining a cigarette was from a store/shop/street hawker followed by other sources such as getting it from someone else/some other way; except for East Timor, where buying from vending machines was the most common method. Similarly, the most preferred methods of obtaining cigarettes among girls were from other sources than from stores/shops/street hawkers; except in East Timor, where vending machines, followed by stores/shops/street hawkers, were the favored methods of buying cigarettes.

The proportion of currently smoking boys who had obtained their cigarettes from a store/kiosk/shop was highest in Bangladesh (85.6%) and lowest in East Timor (20.1%), whereas “other” sources of obtaining cigarettes among currently smoking boys were highest in Sri Lanka (80.3%) and lowest in East Timor (0.5%). Boys getting their cigarettes from the vending machine were observed only in East Timor (79.4%) and Indonesia (0.3%). The proportion of currently smoking girls who had obtained from the store/kiosk/shop was highest in Thailand (65.2%) and lowest in Sri Lanka (0.01%), whereas “other” sources of obtaining cigarettes among currently smoking girls were highest in Bangladesh (100%) and lowest in East Timor (1.3%). Girls getting their cigarettes from the vending machine were observed only in East Timor (69.3%) and Indonesia (1.3%).

**Figure 3:**
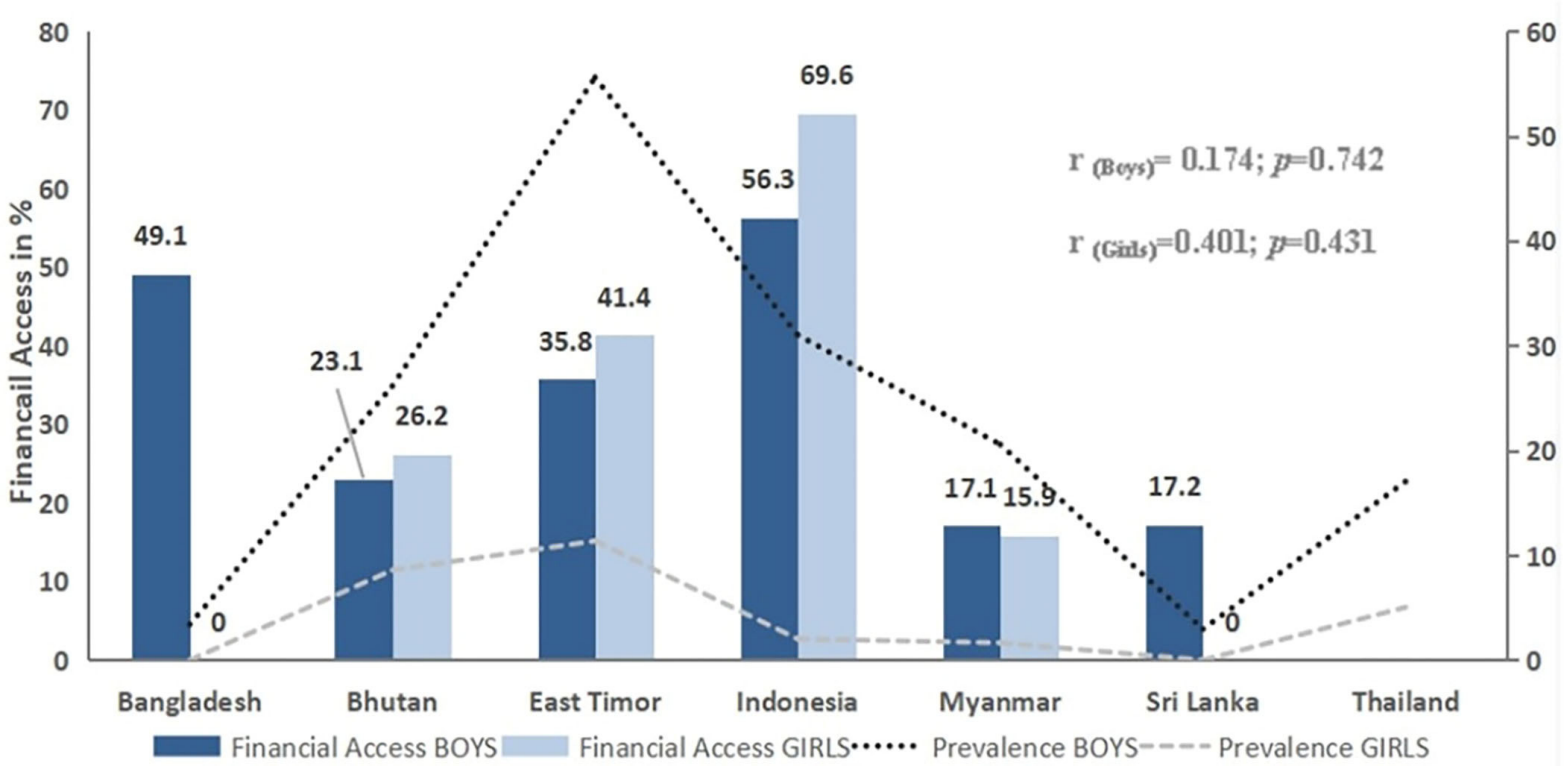
Financial access and prevalence of cigarette smoking among school-going children in seven SEA member countries.

Data on financial access were available for all countries except for Thailand. Except for Indonesia and in all other countries, the pocket money for 7 days was inadequate to purchase a pack of cigarettes for most of the students. Girls' cigarette smoking was more than 2% in Bhutan, East Timor, and Indonesia, and financial access among girls was seen as higher as compared with boys. There was a positive but weak (statistically insignificant) correlation between financial access and cigarette use among boys and girls (Figure 3).

The review of the legal framework suggests that Bangladesh, Bhutan, East Timor, Myanmar, Sri Lanka, and Thailand ratified WHO FCTC in 2003 and 2004. Between 2005 and 2017, the countries enacted their national tobacco control legislation and school tobacco control policies, while GYTS was implemented between 2013 and 2016. East Timor and Thailand lacked tobacco control legislation at the time of GYTS, including a prohibition on tobacco sales to or by minors and vending machines. Despite limits on tobacco sales to minors in other countries, vendors supplied cigarettes to the majority of students, with the highest percentages for Bangladeshi boys (87%) and Indonesian girls (58.1%) (Table 1).

**Table 1:** Tobacco control (TC) policy environment for tobacco use restriction among youths and access to cigarettes by youths in seven South-East Asia member countries as per the Global Youth Tobacco Survey.

**Country** **(GYTS Year)**	**Bangladesh (2013)**	**Bhutan (2013)**	**East Timor (2013)**	**Indonesia (2014)**	**Myanmar (2016)**	**Sri Lanka (2015)**	**Thailand (2015)**
WHO FCTC signatories and ratification	14-06-04	23-08-04	22-12-04	–	21-04-04	11-11-03	08-11-04
Entry into force	27-02-05	27-02-05	22-03-05	–	27-02-05	27-02-05	27-02-05
First school tobacco control policy	2005	2010	2016	2009	2006	2006	2017
Prohibition of sales to or by minors& tobacco vending machines	2005	2010	2016	2012	2006	2015	2017
Legal age forsale or purchase of tobacco	18 Years	18 Years	17 Years	18 Years	18 Years	21 Years	18 Years
**Vendor denied cigarette due to age**
Boys (%)	13	50.9	51.8	38.4	33.2	67.7	61
Girls (%)	0	52.0	48.1	41.9	48.4	0	46.4

## Discussion

In this study, the overall prevalence of current cigarette smoking among youth ranges from nil (girls in Sri Lanka) to 55.57% (boys in East Timor). A comparison of all available data is that the prevalence of current cigarette smoking is increasing in Bhutan, Indonesia, and Myanmar, while it is decreasing in Sri Lanka only. This study finds a higher prevalence of cigarette smoking among boys and girls in East Timor as compared with the other six countries (18). A study in East Timor also showed similar findings that the overall relevance of cigarette smoking among youth is 51% with the rate among boys at 59% and girls at 28%, respectively (19). The purchase of cigarettes from a store, shop, or street vendor was by far the most preferred method, followed by purchases made from other sources, such as friends or colleagues, or other methods. Evidence suggests that underage prohibitions in SEA member countries are not well-enforced, and adolescents have relatively easy access to tobacco ***via*** shops and stores (20). Despite insufficient pocket money, youths have easy access to cigarettes. The review of existing studies indicates that SEA member countries have not yet fully implemented and/or enforced laws that would make tobacco products less affordable and accessible (21). These laws include taxes, the sale of single cigarettes or loose tobacco products, and minimum legal smoking ages (22).

Framework Convention on Tobacco Control has been in place since 2004 to flatten the tobacco pandemic curve. Article 6 (tobacco taxation), Article 13 (ban on tobacco advertisements, promotions, and sponsorship), and the FCTC demand control steps have the propensity to protect the youths from tobacco use (23, 24). In addition, supply reduction by eliminating the illegal trade of tobacco products (Article 15) and restricting the selling of tobacco products to and by minors (Article 16) have also been implicated in minimizing the number of youth smokers (25).

Varying levels of enforcement of tobacco control policies across the SEA region might be the reason for the differential prevalence of cigarette smoking and access to cigarettes by youths. Youths' cigarette purchase is highly price-sensitive, which makes them vulnerable to less expensive purchases; getting is a blessing in disguise due to the availability of single sticks for the purchase of cigarettes, which is familiar in some countries (16). Full implementation of Article 16 faces critical barriers in the form of the attempt by the tobacco industry to undermine access laws, retailers' opposition, incomplete enforcement, and access to cigarettes at unregulated alternative outlets (17).

The vulnerable youth population was the victim of the higher prevalence of smoking due to the economic growth and the presence of a stronger tobacco industry in the region (26). Furthermore, the definition of the legal age for youths in buying cigarettes by country-specific tobacco control legislation varies. Despite the ban, most students can get their cigarettes from stores or shops or kiosks with limited objection from the vendors that are a cause of concern (27).

The differential tobacco use burden across countries in the SEA region and genders within countries, following full implementation of comprehensive tobacco control measures, may be the result of contextual sociocultural norms and adult tobacco use (28). The projected one billion tobacco global fatalities in this century (15) can be effectively countered by the successful implementation of various provisions of FCTC, especially Article 16 (29). To curtail the factors affecting youth tobacco use, dissuading illegal sales to those under 18 years of age should be strictly enforced with existing anti-tobacco laws. Anti-tobacco legal provisions are effective only when comprehensive tobacco control measures are in place (30).

### Limitations

Self-reported data from 13 to 15 years of school-going students may be subjected to misreporting and may not be representative of the entire youth community in the given country. The GYTS survey years 2013–2016 may limit describing recent smoking behavior.

## Conclusion

Youth access to cigarettes is highly contextual and can defy legal restrictions and financial affordability. Boys consistently outnumbered their girl counterparts in smoking tobacco use emphasizing the need for an additional high-risk tobacco control approach for boys. Comprehensive tobacco control policies aimed at limiting youth access to tobacco products should be studied in the context of the respective country's social-cultural, financial, and regulatory surroundings. Tobacco promoter's activity needs to be linked with youth's access to tobacco products. The Global Youth Tobacco Survey may be routinely implemented to monitor tobacco use among youths and the effectiveness of the tobacco control policy.

### Implications for policy and practice

Evidence suggests that interventions such as limiting access to tobacco products can successfully be implemented only if comprehensive tobacco control measures such as taxation, health warnings, and bans are in place. Understanding how and where youths have access to cigarettes can help in devising effective tobacco control strategies. Restricting access and age restrictions on tobacco product sales have been enforced in many countries with varying success, due to resource constraints that inhibit the implementation of these laws. Access to cigarettes among youths is very contextual and can defy legal provisions and financial affordability. Comprehensive tobacco control strategies aimed at restricting youths' access to tobacco products may be viewed in the context of country-specific socio-cultural, economic, and legal environments.

## Data availability statement

Publicly available datasets were analyzed in this study. This data can be found here: https://www.cdc.gov/tobacco/global/gtss/gtssdata/index.html.

## Ethics statement

Ethical review and approval was not required for the study on human participants in accordance with the local legislation and institutional requirements. Written informed consent from the (patients/participants OR patients/participants legal guardian/next of kin) was not required to participate in this study in accordance with the national legislation and the institutional requirements.

## Author contributions

NS, PJ, and VE equally contributed to the study design, data analysis, and interpretation. NS made the first draft. PJ and VE have critically reviewed with substantial contributions to the content. All authors participated in the conceptualization of the study, reviewed, and approved the manuscript.

## Conflict of interest

The authors declare that the research was conducted in the absence of any commercial or financial relationships that could be construed as a potential conflict of interest.

## Publisher's note

All claims expressed in this article are solely those of the authors and do not necessarily represent those of their affiliated organizations, or those of the publisher, the editors and the reviewers. Any product that may be evaluated in this article, or claim that may be made by its manufacturer, is not guaranteed or endorsed by the publisher.
